# AZD9291 Resistance Reversal Activity of a pH‐Sensitive Nanocarrier Dual‐Loaded with Chloroquine and FGFR1 Inhibitor in NSCLC

**DOI:** 10.1002/advs.202002922

**Published:** 2020-12-04

**Authors:** Yu Gu, Songtao Lai, Yang Dong, Hao Fu, Liwei Song, Tianxiang Chen, Yourong Duan, Zhen Zhang

**Affiliations:** ^1^ Department of Radiation Oncology Fudan University Shanghai Cancer Center Shanghai 200032 China; ^2^ Department of Oncology Shanghai Medical College Fudan University Shanghai 200032 China; ^3^ State Key Laboratory of Oncogenes and Related Genes Shanghai Cancer Institute Renji Hospital School of Medicine Shanghai Jiao Tong University Shanghai 200032 China; ^4^ Shanghai Lung Cancer Center Shanghai Chest Hospital Shanghai Jiao Tong University Shanghai 200030 China

**Keywords:** autophagy, AZD9291, chloroquine, fibroblast growth factor receptor 1, nanoparticles

## Abstract

AZD9291 can effectively prolong survival of non‐small cell lung cancer (NSCLC) patients. Unfortunately, the mechanism of its acquired drug resistance is largely unknown. This study shows that autophagy and fibroblast growth factor receptor 1 signaling pathways are both activated in AZD9291 resistant NSCLC, and inhibition of them, respectively, by chloroquine (CQ) and PD173074 can synergistically reverse AZD9291 resistance. Herein, a coloaded CQ and PD173074 pH‐sensitive shell–core nanoparticles CP@NP‐cRGD is developed to reverse AZD9291 resistance in NSCLC. CP@NP‐cRGD has a high encapsulation rate and stability, and can effectively prevent the degradation of drugs in circulation process. CP@NP‐cRGD can target tumor cells by enhanced permeability and retention effect and the cRGD peptide. The pH‐sensitive CaP shell can realize lysosome escape and then release drugs successively. The combination of CP@NP‐cRGD and AZD9291 significantly induces a higher rate of apoptosis, more G0/G1 phase arrest, and reduces proliferation of resistant cell lines by downregulation of p‐ERK1/2 in vitro. CQ in CP@NP‐cRGD can block protective autophagy induced by both AZD9291 and PD173074. CP@NP‐cRGD combined with AZD9291 shows adequate tumor enrichment, low toxicity, and excellent antitumor effect in nude mice. It provides a novel multifunctional nanoparticle to overcome AZD9291 resistance for potential clinical applications.

## Graphical abstract

This work develops a coloaded chloroquine and fibroblast growth factor receptor 1 (FGFR1) inhibitor pH‐sensitive shell–core nanoparticles CP@NP‐cRGD, which can significantly dual‐block autophagy and FGFR1 pathway to overcome AZD9291 resistance. CP@NP‐cRGD can ensure excellent tumor targeting ability, realize lysosomal escaping, and release drugs sequentially by pH‐sensitive CaP shell. CP@NP‐cRGD has low toxicity and long circulation time in vivo.

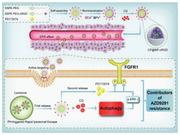

## 1 Introduction

Non‐small cell lung cancer (NSCLC) accounts for ≈80–85% of lung cancer cases,^[^ ^1^ ^]^ with a 5‐year survival rate of only 21%.^[^ ^2^ ^]^ In the past 15 years, with the deepening understanding of the mechanism of epidermal growth factor receptor (EGFR) mutation in NSCLC, oral tyrosine kinase inhibitors (TKIs) targeting EGFR were developed, which changed the situation of NSCLC treatment.^[^ ^3^ ^]^ For the 1st and 2nd generation TKI, although the response rates were not low, acquired drug resistance would be developed within one year.^[^ ^4^ ^]^ AZD9291 is the first 3rd‐generation EGFR‐TKI approved by the European Medicines Agency and the United States Food and Drug Administration (FDA), which not only has a noticeable effect on classical EGFR sensitizing mutation (exon 18, 19, 21) but also can inhibit EGFR T790M mutation, the most frequent resistance mechanism of the 1st and 2nd generation TKI.^[^ ^5^ ^]^ AZD9291 has effectively prolonged the survival of NSCLC in several phase III studies; even so, acquired resistance of it also inevitably occurs and hinders its benefit.^[^ ^6^ ^]^ Despite increasing reports of AZD9291 for NSCLC treatment, only limited data from individual cases or clinical series reported its resistance‐related mechanisms such as C797S mutation.^[^ ^7^ ^]^ There are two primary mechanisms involved in the EGFR‐TKI resistant process: target gene alteration and EGFR‐independent signaling pathways.^[^ ^8^ ^]^ In comparison with EGFR mutations, the mechanisms associated with acquired resistance involving signaling pathways without genetic changes have been less reported in the literature.

Autophagy, a highly conserved cellular process by which cytosolic proteins and organelles are recycled to replenish intracellular nutrients and energy, is more and more accepted as a protective mechanism for cells to adapt to stressful conditions.^[^ ^9^ ^]^ Recent evidence suggests that autophagic pathways enable cancer cells to enhance survival at late‐stage or under therapeutic stresses.^[^ ^10^ ^]^ Since autophagy has a context‐dependent role in malignant tumor development, inhibition of autophagy appears to be a promising strategy to decrease AZD9291 resistance. As the only clinically‐approved autophagy inhibitor, chloroquine (CQ) was first widely used in the 1940s as an antimalaria drug due to its efficiency and cost‐efficient synthesis.^[^ ^10^, ^11^ ^]^ CQ can block the fusion of autophagosomes with lysosomes and result in the inhibition of lysosomal degradation. This process appears in three autophagic pathways in mammalian cells, including macroautophagy, microphagy, and chaperone‐mediated autophagy, despite different mechanisms for delivery of cargo to lysosomes.^[^ ^12^ ^]^ Furthermore, CQ can act as an effective agent to reduce the lysosomal trapping of anticancer drugs such as TKI from the lysosome.^[^ ^13^ ^]^ At present, CQ and its derivatives are undergoing evaluation of several clinical cancer trials when combined with other medications.^[^ ^14^ ^]^ However, the limited efficacy and systemic toxicity of these agents make it urgent to develop better methods targeting autophagy in cancer cells.^[^ ^15^ ^]^

Many receptor tyrosine kinases (RTKs) have been identified in NSCLC tumor samples and may act as a bypass activation of the EGFR signaling pathway in NSCLC, such as Met and IGF‐1R.^[^ ^16^ ^]^ Previous studies have shown that fibroblast growth factor receptor 1 (FGFR1), a typical RTKs, is overexpressed in various types of human cancers, including 20% of NSCLC. It participated as a driver in tumorigenesis, tumor progression, and chemoresistance.^[^ ^17^ ^]^ Also, FGFR1 signaling was described to contribute to epithelial‐to‐mesenchymal transition (EMT)‐associated acquired resistance of 1st and 2nd EGFR‐TKIs,^[^ ^18^ ^]^ which provided a bypass protective mechanism after prolonged exposure to pharmacological‐inhibited EGFR signaling. However, only very few reports have described the role of the FGFR1 pathway in AZD9291 resistance. Since several preclinical and clinical data have shown that NSCLC cells with acquired 1st or 2nd EGFR‐TKI resistance to be sensitive to FGFR1 inhibitors, the FGFR1 blockade could also be a promising clinical strategy to overcome AZD9291 resistance.^[^ ^17^, ^18^, ^19^ ^]^ Importantly, studies have suggested that FGFR signaling regulates autophagy during processes of disease progression and treatment.^[^ ^20^ ^]^ FGFR1 TKI induces protective autophagy by inhibiting AKT/mTOR signaling pathway in FGFR1‐amplified breast cancer cell, and autophagy inhibition could further enhance the anticancer effects of FGFR1 inhibitor.^[^ ^21^ ^]^ These results indicated that the FGFR1 pathway had an inhibitory effect on autophagy, targeting both the FGFR1 pathway and autophagy might be a new turning point in AZD9291 treatment.

**Figure 1:**
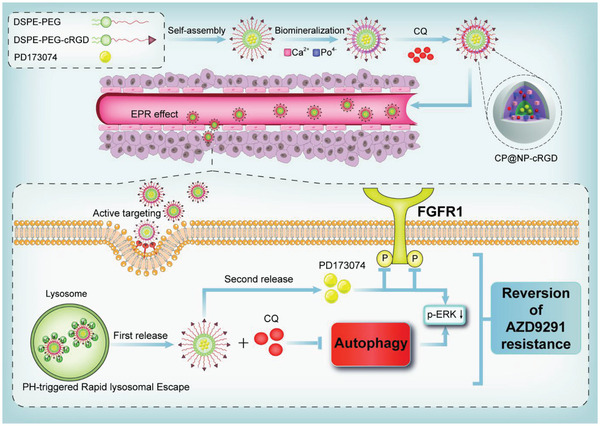
The schematic illustration of the formation process and intracellular mechanism of the CP@NP‐cRGD. CP@NP‐cRGD was prepared by biomineralization method. PD173074 was first encapsulated with organic DSP‐PEG‐CRGD core, and then CQ was adsorbed in the inorganic CaP shell. The working mechanism of CP@NP‐cRGD in NSCLC cells is as follows: (a) the appropriate size of nanoparticles results in the EPR effect, (b) cRGD short peptide mediates the enhanced active tumor targeting effect, (c) pH‐sensitive CaP shell causes lysosomal escape, (d) CQ is firstly released to inhibit autophagy, (e) then PD173074 is released to inhibit FGFR1 pathway, and (f) inhibition of the above pathways results in down‐stream pERK downregulation, which synergistically reverses AZD9291 resistance.

In this study, we investigated the potential role of dual‐targeting FGFR1 pathway and autophagy in overcoming NSCLC AZD9291 resistance. First, we found that AZD9291‐resistant cells showed higher autophagy and FGFR1 expression level compared with AZD9291‐sensitive cells. AZD9291 induced autophagy and up‐regulated FGFR1 expression in AZD9291‐resistant cells. We also found that inhibiting the FGFR1 pathway could upregulate autophagy in AZD9291‐resistant cells. Currently, a combination of various drugs of different targets has proved to be a sound strategy for cancer therapy. The experience of epidemiologists underscores the importance of rapid, effective, and a combination of drug use in preventing tuberculosis and HIV drug resistance.^[^ ^22^ ^]^ We hypothesize that dual FGFR1‐autophagy blockade as a mechanism‐based combination may be a promising clinical strategy to reverse AZD9291 resistance. The safety and efficiency of the complex agent are fundamental premises for application. Here, we presented multifunctional tumor‐targeted nanoparticles (NPs) that coloaded with autophagy inhibitor CQ and selective FGFR1 inhibitor PD173074. Compared with inhibition of FGFR1 or autophagy alone, inhibition of both pathways at the same time can effectively improve drug resistance of AZD9291. Also, our results showed that inhibition of the FGFR1 pathway and autophagy significantly reduced the kinase activity of ERK1/2. The pH‐sensitive NPs constructed by us have high encapsulation rate and stability and can effectively prevent the degradation of CQ and PD173074 in the circulation process, ensuring that they could achieve the drug activity within the tumor tissue. Besides, NPs delivered drugs to tumor tissue by enhanced permeability and retention (EPR) effects and actively targeted tumor cells by the cRGD peptide on the shell.^[^ ^23^ ^]^ The pH‐sensitive CaP shell dissolved in the lysosome and disintegrates the lysosomal membrane,^[^ ^24^ ^]^ realizing drug escape from the lysosome. At last, the NPs released drugs successively and sustainably. It first released CQ to reduce the autophagy of tumor cells, and then released PD173074 to inhibit FGFR1 activity. The multifunctional NP CP@NP‐cRGD has outstanding tumor targeting, high efficiency, low toxicity, and sustained release properties, which provides a new prospect for overcoming AZD9291 resistance (**Figure** **1**).

## 2 Results

### 2.1 The Increased Level of Autophagy and FGFR1 Expression in AZD9291‐Resistant Cells with or without AZD9291

**Figure 2:**
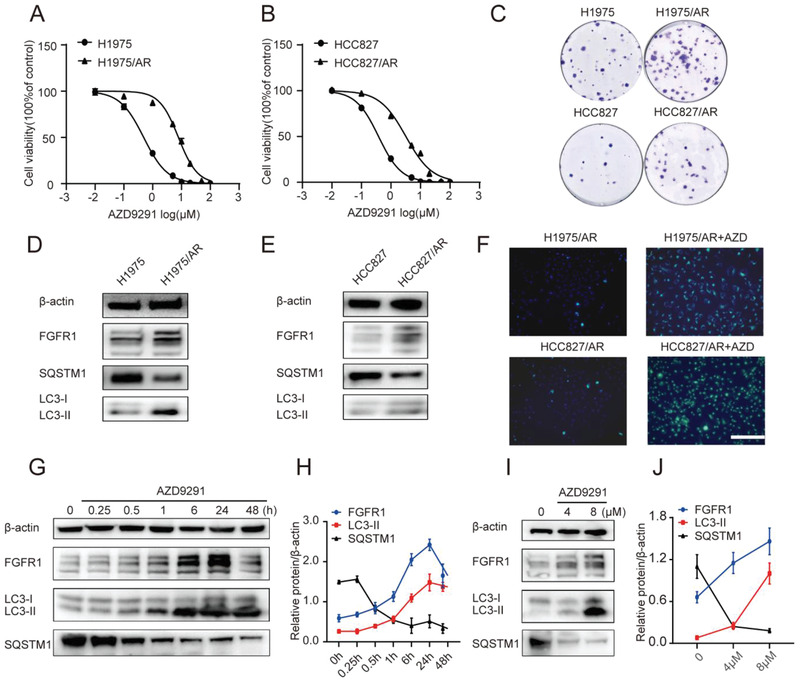
The increased level of autophagy and FGFR1 expression in AZD9291‐resistant cells with or without AZD9291. A,B) Acquisition of H1975 and HCC827 cells resistant to AZD9291. The drug sensitivity of parental and AZD9291‐resistant cells was detected by CCK8 assay. IC50 analysis was performed using GraphPad analysis software. C) Representative colonies of parental and AZD9291‐resistant cells treated with AZD9291 for 48 h before clonogenic assay. D,E) Western blotting showing the expression levels of FGFR1 and autophagy‐related protein in parental and AZD9291 resistant cells. F) Autophagy was monitored based on MDC staining 48 h post‐AZD9291 treatment. Scale bar: 100 µm. G–J) Western blotting showing increase level of FGFR1 and LC3‐II, decrease level of SQSTM1 in H1975/AR cells treated with AZD9291 during the increasing time course (G,H) or with increasing concentrations (I,J). Quantitative grayscale intensity of protein expression was performed using ImageJ software. Values were normalized to ß‐actin protein levels. All data were from three repeats. IC50: half maximal inhibitory concentration.

In order to explore the mechanisms of acquired resistance to AZD9291, AZD9291‐resistant NSCLC cell lines (H1975/AR and HCC827/AR) were established from the parental H1975 (EGFR L858R + T790M) and HCC827 (EGFR E746‐A750del) AZD9291‐sensitive cell line respectively by gradually increasing concentrations of AZD9291, as described in Methods. The half‐maximal inhibitory concentration value (IC50) of AZD9291 for H1975/AR cells was 7.84 µmol, which was nearly 16‐fold higher than that for the H1975 cells (0.49 µmol) (**Figure** **2**A). And the IC50 values of AZD9291 for HCC827 and HCC827/AZDR were 0.38 and 3.21 µmol, respectively (Figure 2B). The clonogenic ability of AZD9291‐resistant cells was significantly enhanced compared with that of sensitive cells in H1975 and HCC827 cells (*P* < 0.05) (Figure 2C). Results showed that the expression of FGFR1 protein was higher in H1975/AR and HCC827/AR cell compared with parental cell lines (Figure 2D,E; Figure S1, Supporting Information). The endogenous levels of autophagy were also compared between AZD9291‐resistant and AZD9291‐sensitive cell lines by detecting the protein expression levels of autophagy‐related protein LC3B and SQSTM1. During autophagy, cytoplasmic LC3 (LC3‐I) is transformed into autophagosome membrane LC3 (LC3‐II), leading to increased levels of LC3‐II. SQSTM1 is an autophagy substrate degraded by lysosomes, so the level of SQSTM1 always decreases during autophagy. H1975/AR and HCC827/AR cells showed higher protein expression levels of LC3‐II and lower levels of SQSTM1, comparing with H1975 and HCC827 cells, respectively (Figure 2D,E; Figure S1, Supporting Information). The above results suggested that background autophagy and FGFR1 levels of drug‐resistant cells were higher than those of sensitive cells, and these two factors may be related to the self‐protection mechanism of lung cancer cells that cause AZD9291 resistance. To further demonstrate whether AZD9291 treatment activates these two pathways, we added AZD9291 to drug‐resistant cells to observe FGFR1 protein and autophagy levels. Dansylcadaverine (MDC) staining assay was used to observe autophagic vacuoles, which could specifically bind to autophagic vacuoles. The fluorescence intensity of MDC staining significantly enhanced with AZD9291 treatment, suggesting an increased autophagy level (Figure 2F).

Also, as shown in Figure 2G–J, AZD9291 regulated FGFR1 and autophagy‐related protein levels in a dose‐ and time‐dependent manner. Collectively, these data demonstrated that AZD9291 resistance might be associated with FGFR1 overexpression and autophagy activation.

### 2.2 Blocking of FGFR1 Inhibitor Induced Autophagy by CQ Could Significantly Enhance the Reversion of AZD9291 Resistance

**Figure 3:**
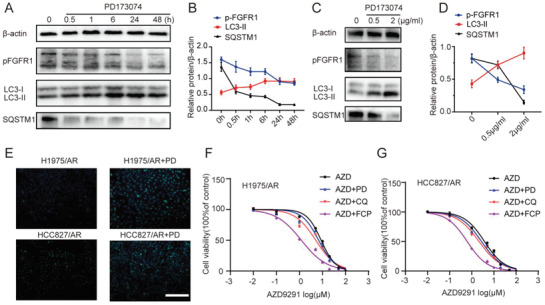
CQ blocking PD173074 induced autophagy could significantly enhance the reverse of AZD9291 resistance. A–D) Western blotting analysis showing decrease in the level of p‐FGFR1 and SQSTM1, increase in the level of LC3‐II in H1975/AR cells treated with PD173074 during the indicated time course (A,B) or with increasing concentrations (C,D). Quantitative grayscale intensity of protein expression was performed using ImageJ software. Values were normalized to ß‐actin protein levels. E) Autophagy was monitored based on MDC staining 48 h post‐PD173074 treatment. Scale bar: 100 µm. F,G) The drug sensitivity of AZD9291‐resistant cells treated with AZD9291, AZD9291 + CQ, AZD9291 + PD173074, and AZD9291 + CQ + PD173074 were detected by CCK8 assay. All data were from three repeats. CQ: chloroquine, PD: PD173074, FCP: free chloroquine and PD173074.

Our results showed that the selective FGFR1 inhibitor PD173074 could significantly decrease the protein level of pFGFR1 at the concentrations of 0.5 µg mL^−1^, suggesting the effective inhibition of the FGFR1 pathway in AZD9291 resistant NSCLC cells (**Figure** **3**A). Considering the cytotoxicity and inhibitory activity of PD173074, 0.5 µg mL^−1^ was selected as the optimal concentration for subsequent experiments (Figure S2A, Supporting Information). FGFR1 has also been reported to stimulate autophagy^[^ ^25^ ^]^ and we further explored their relationship in AZD9291‐resistant cell lines. We found that the protein level of autophagy‐related markers LC3‐II increased, and SQSTM1 decreased in a time‐dependent and dose‐dependent manner (Figure 3A–D). The fluorescence intensity of MDC staining was also enhanced after PD173074 treatment (Figure 3E). The sensitivity to AZD9291 was restored when cells were treated with the combination of PD173074 and AZD9291. Autophagy inhibitor CQ combined with PD173074 showed a significantly better drug resistance reversal effect in AZD9291‐resistant when compared with either agent alone (Figure 3F,G). The cytotoxicity of CQ at 10 µg mL^−1^ was very low, and autophagy could be effectively inhibited, so the concentration was used for subsequent experiments (Figure S2B, Supporting Information).

### 2.3 Synthesis and Characterization of CP@NP‐cRGD

It is well known that RGD peptide (arginine–glycine–aspartic) has a high affinity for cell adhesion receptor integrin, which plays a vital role in tumor angiogenesis.^[^ ^26^ ^]^ NPs can actively target tumor cells through the modification of circular RGD (cRGD). We prepared the cRGD‐functionalized PEG‐DSPE by the amidation reaction between DSPE‐PEG_2k_‐COOH and cRGD. The successful synthesis and chemical structure of cRGD‐PEG‐DSPE was confirmed by ^1^H‐NMR (Figure S3A,B, Supporting Information). PEG is approved by FDA, so cRGD‐PEG‐DSPE is considered to be a biocompatible carrier material with low toxicity to the human body. We prepared CP@NP‐cRGD containing CQ and PD173074 by a biomineralization method with good repeatability. In simple terms, we used hydrophobic DSPE to load PD173074 to form an interior core, and used hydrophilic cRGD‐PEG to entrap CQ with calcium phosphate (CaP) to form a shell.^[^ ^27^ ^]^

**Figure 4:**
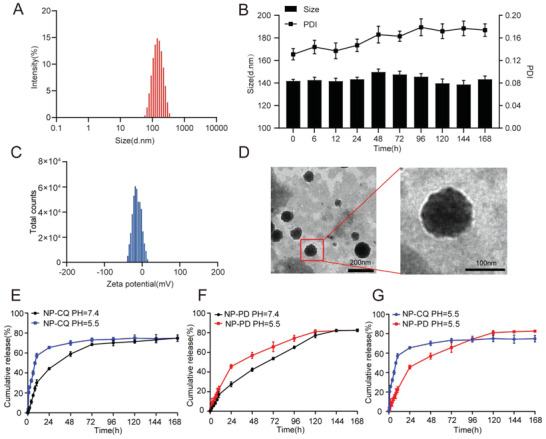
Characterization of CP@NP‐cRGD. A) Typical particle size distribution diagrams of CP@NP‐cRGD with optimized formulation. B) The physical stability of CP@NP‐cRGD at 37 °C in serum (5%, pH 7.4). The hydrodynamic size and PDI of CP@NP‐cRGD were determined over one week. C) The average potential of CP@NP‐cRGD. D) Transmission electron microscopy image of CP@NP‐cRGD. E,F) *In vitro* release profile of CQ (E) and PD173074 (F) from CP@NP‐cRGD with the dialysis bag diffusion technique in PBS at pH 5.5 and 7.4. G) CQ and PD173074 were sequentially released from NPs in pH 5.5. Each data was expressed as mean ± standard deviation (*n* = 3).

The sizes and zeta potential distributions of NPs were presented in **Figure** **4**A–C, respectively. The average size of CP@NP‐cRGD was measured as 123.4 ± 0.4 nm with a polydispersity index (PDI) of <0.25 and could contribute to a relatively good EPR effect. CP@NP‐cRGD in aqueous solution was negatively charged with a zeta potential of −15.1 ± 1.4 mV, which could keep the stability of targeted NPs through the electrostatic repulsion. The long‐term stability of CP@NP‐cRGD at 37 °C in 5% serum (pH 7.4) can be estimated by examining the fluctuation of particle size (Figure 4B). The average particle size and PDI of CP@NP‐cRGD fluctuated in a small range within a week, indicating that there was no obvious aggregation and precipitation. The relative good long‐term stability in serum suggested that CP@NP‐cRGD was suitable for in vivo experiments. The drug encapsulation rate (EE%) and drug loading efficiency (DL%) of CQ were 73.7% ± 0.6% and 4.6% ± 0.9%, respectively. The EE% and DL% of PD173074 were 84.6% ± 2.6% and 0.3% ± 0.1%, respectively. Transmission electron microscopy (TEM) was utilized to study the morphology of CP@NP‐cRGD (Figure 4D). The results of TEM showed that the NPs were self‐assembled spherical vesicles with uniform particle size and high dispersion characteristics.

PH‐sensitive CaP‐NPs can keep the drug be stable at normal physiological pH value and sensitive to the low pH value of the tumor environment. They may realize lysosomal escape and disintegrate in an acidic microenvironment that leads to the burst release of CQ in the shell before PD173074 in the core. In vitro drug release of CP@NP‐cRGD was studied at different pH values, as shown in Figure 4E–G. Results showed that CQ and PD173074 in NPs released drugs faster at pH 5.5 than at pH 7.4 (*p* < 0.05). The pH‐triggered drug release pattern reduces the exposure and toxicity of the drug in circulation (pH 7.4), allowing the rapid release of the drug from the NPs after endocytosis. The release behavior of CQ in NPs is considered as the biphasic release with initial blasting effect, followed by a sustained release as depicted in Figure 4E. The release amount of CQ was 57.2% at pH 5.5, and 30.1% at pH 7.4 at 10 h, and then the curve tended to flatten out beyond this. PD173074 showed a relatively slow phase over periods. In addition, unlike the nanodelivery system, the release curves of free drugs have no prolonged‐release properties and no sequential release ability in acidic environments (Figure S4A–C, Supporting Information), which highlights the unique drug release advantage of CP@NP‐cRGD.

### 2.4 CP@NP‐cRGD Resensitizes NSCLC to AZD9291 In Vitro

**Figure 5:**
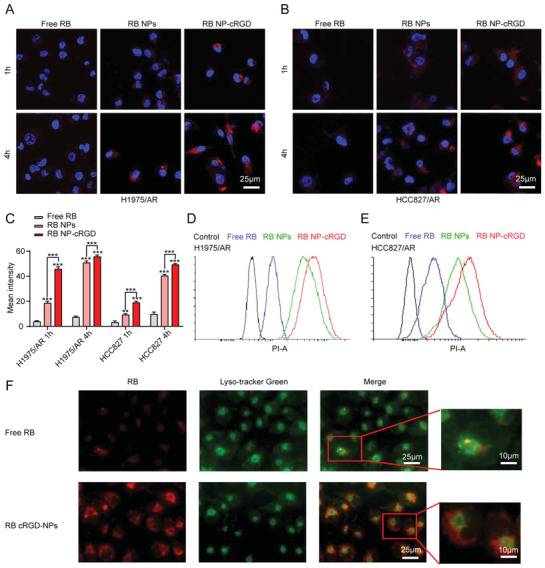
Cellular uptake and lysosome escape of CP@NP‐cRGD in vitro. A,B) Confocal microscopy observed the cellular uptake of NPs in H1975/AR and HCC827/AR cells (At scale 25 µm). Cells were treated with free RB, RB NPs, RB NP‐cRGD for 1 and 4 h. All images showed merged images, including RB (red) and nuclei (blue). C) Quantitative fluorescence intensity of RB in the AZD9291‐resistant cells. The internalized RB ranged from more to less: RB NP‐cRGD > RB NPs > free RB. Note: **p < 0.01, ***p < 0.001, compared with free RB group. D,E) Flow cytometry analyzed the cellular uptake of NPs in H1975/AR and HCC827/AR cells at 4 h. F) The lysosomal escape of NP‐cRGD after 2 h uptake in H1975/AR cells. All data are from three repeats. RB: Rhodamine B.

To examine the effect of NPs on AZD9291 resistance in vitro, first, we examined the toxicity of cRGD‐NPs in AZD9291 resistant cell lines. After incubation with different concentrations of cRGD‐NPs for 72 h, the survival rates of H1975/AR cells and HCC827/AR cells were not significantly decreased, indicating that the cytotoxicity of cRGD‐NPs was very low (Figure S5A,B, Supporting Information). Next, the uptake capacity of NPs was detected. Rhodamine B (RB) was encapsulated in the NPs and the uptake of NPs in H1975/AR cells, and HCC827/AR cells was observed, as shown in **Figure** **5**A,B. The orders of fluorescence intensity from strong to weak were as follows: free RB group < NPs‐RB < cRGD‐NPs‐RB, indicating that NPs can significantly enhance translocation drugs into cells and cRGD moieties can facilitate high affinity to integrin *α*v*β*3/*α*v*β*5‐rich cancer cells such as H1975 and HCC827 (Figure 5C; Figure S6, Supporting Information).^[^ ^28^ ^]^ The cellular uptake was also investigated by flow cytometry. The flow cytometry showed that the mean fluorescence intensity of cRGD‐NPs group was stronger than NPs group (Figure 5D,E, Supporting Information). Moreover, the fluorescence intensity of RB at 4 h was higher than that at 1 h, indicating that the internalization of NPs was time‐dependent.

The passage from lysosomes into the cytoplasm is considered to be a critical step for drug delivery. Lysosomes are membrane‐bound organelles with an internal pH of about 5.5 and contain enzymes that inactivate the drugs. Under acidic conditions, the CaP shell will crack to form ion pairs with the lysosome membrane and increase the osmotic pressure of lysosomes, resulting in lysosome rupture. The overlapping red fluorescence and lysosomal green fluorescence of the free drug group suggested that most free drugs were captured in lysosomes. By contrast, the apparent separation of red fluorescence indicated a successful lysosomal escape of the NPs group after 2 h uptake (Figure 5F).

**Figure 6:**
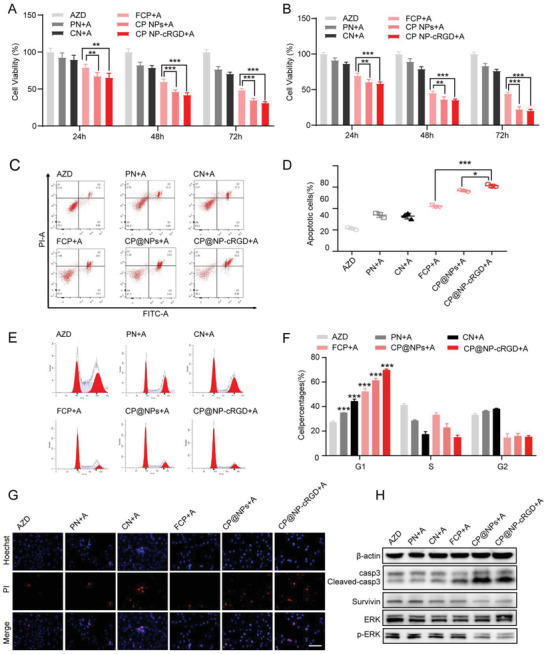
Combination of CP@NP‐cRGD and AZD9291 enhances the antitumor effect in vitro. A,B) Cell viability of H1975/AR and HCC 827/AR cells with various treatments for 72 h by CCK8 assay. C) Apoptosis of H1975/AR cells in vitro determined by flow cytometric analysis with Annexin‐V‐FITC and PI double‐staining after incubated with different drugs for 48 h. D) The percentage of apoptotic cells included early apoptotic cells and late apoptotic cells. E,F) Flow cytometric analysis evaluated cell cycle distribution. Representative histograms shown in (E) and quantitative histograms shown in (F). The percentage of cells in each cell cycle phase in different groups was compared with AZD9291 alone group. G) Images showing the effect of different drugs of H1975/AR detected with dual staining of Hoechst 33324/PI. Blue color represented being stained with Hoechst, apoptotic cells appeared strong fluorescent signals, and healthy cells showed weakly stained. Red color represented PI staining indicating necrotic cells. Scale bar: 50 µm. H) Western blotting images showing the expression level of apoptosis‐associated and ERK proteins. All data were from three repeats, *p < 0.05, **p < 0.01, ***p < 0.001. A: AZD9291, AZD: AZD9291, PN: PD@NP‐cRGD, CN: CQ@NP‐cRGD, FCP: free CQ and PD173074.

To overcome autophagy and FGFR1‐overexpression induced resistance, we investigated whether dual‐target CP@NP‐cRGD can reverse or overcome acquired EGFR‐TKI resistance. To evaluate the antiproliferative effects of the combination of AZD9291 with free CQ/PD(FCP), CQ@NP‐cRGD(CN), PD@NP‐cRGD(PN), CP@NPs, or CP@NP‐cRGD, we have conducted the CCK8 studies. After 72h treatment, the cells viability of H1975/AR and HCC827/AR cells was significantly lower in CP@NPs and CP@NP‐cRGD group compared to FCP, CN, and PN group(**Figure** **6**A,B).

To investigate whether drug‐induced cell viability reduction was accompanied by apoptosis or cell cycle arrest, we further performed flow cytometry analysis. As shown in Figure 6C,D, CP@NP‐cRGD group and CP@NPs resulted in 2.87‐fold (61.2% ± 1.5% CP@NP‐cRGD + AZD vs 21.3% ± 1.1% AZD) and 2.65‐fold (56.6% ± 1.1% CP@NPs + AZD vs 21.3% ± 1.1% AZD) increase of apoptosis cells, respectively. In H1975/AR cells, the number of cells with G0/G1 cell cycle arrest was significantly increased in the combination group compared with the AZD9291 alone group (Figure 6E,F). The percentage of AZD9291‐resistant cells in the G0/G1 phase was 27.24% ± 1.13% for the single AZD9291 group, 61.32% ± 1.49% for CP@NPs group, and 70.00% ± 0.86% for the CP@NP‐cRGD group. Normal progression through the G0/G1 phase of the mammalian cell cycle is dependent on the activities of CDK4 and CDK2. As Figure S7 of the Supporting Information shown, our combination treatment inhibited CDK2 and CDK4 protein level, which contributed to G0/G1 arrest. These results revealed that the sensitivity to AZD9291 was restored when cells were treated with the combination. The cell death rate of H1975/AR cells detected by Hoechst 33342/propidium iodide (PI) staining demonstrated that CP@NP‐cRGD enhanced AZD9291‐induced cell death (Figure 6G; Figure S8, Supporting Information).

ERK1/2 pathway is an important intracellular mediator of the EGFR signaling network and is involved in both the FGFR1 and autophagy activation related pathways.^[^ ^29^ ^]^ Therefore, we investigated whether CP@NP‐cRGD affected the activation status of the ERK signaling pathway. In our study, the combination of the CP@NP‐cRGD with AZD9291 showed inactive phosphorylated ERK1/2 and restored the apoptotic pathway as the increased level of cleaved caspase3 and decreased level of survivin (Figure 6H; Figure S9, Supporting Information), suggesting that ERK1/2 pathways participate in the reversed AZD9291 resistance by CP@NP‐cRGD.

Together, these data indicate that simultaneous inhibition of autophagy and the FGFR1 pathway by CP@NP‐cRGD could sharply reverse AZD9291 resistance in NSCLC cell lines.

### 2.5 CP@NP‐cRGD Inhibits Protective Autophagy of AZD9291‐Resistant Cells In Vitro

**Figure 7:**
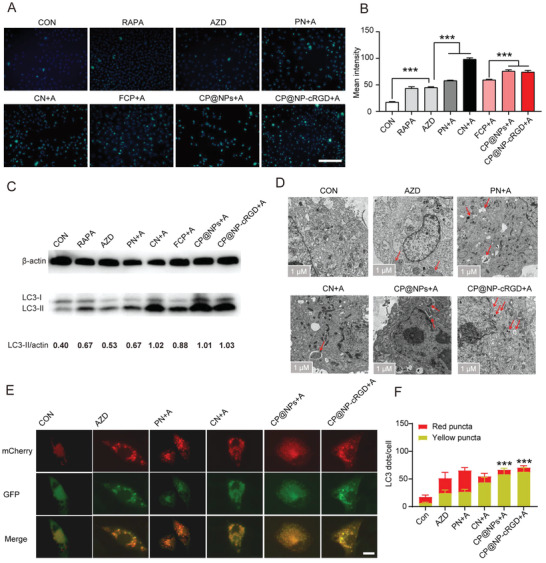
CP@NP‐cRGD inhibits protective autophagy in AZD9291‐resistant cells in vitro A) Fluorescence microscopy images showing autophagic vacuoles with MDC staining in H1975/AR with different treatment for 48 h. Scale bar: 100 µm. B) Quantification of MDC‐positive cells using ImageJ software. C) Western blotting images revealing the expression level of autophagy‐associated proteins LC3‐II. Values were measured using ImageJ software and normalized to ß‐actin protein levels. D) Autophagic ultrastructural features in H1975/AR cell treated with different drugs for 48 h were observed by TEM. Arrows referred to autophagy vesicles. Scale bar: 1 µm. E,F) H1975/AR cells stable expressing mCherry–GFP–LC3B were incubated with different treatments for 24 h. GFP was quenched in lysosomes while mCherry retained the red signal. The autophagosomes and autolysosomes were presented as yellow (mCherry and GFP) and red (mCherry only) puncta, respectively. Representative images were shown in (E), quantitation of autophagosomal/autolysosomal LC3B puncta were shown in (F). Scale bar: 10 µm. All data are from three repeats, *p < 0.05, **p < 0.01, ***p < 0.001. A: AZD9291, PN: PD@NP‐cRGD, CN: CQ@NP‐cRGD, FCP: free CQ and PD173074.

In this part, we further confirmed the crucial role of autophagy in AZD9291 resistance and the function of CP@NP‐cRGD on autophagy regulation. CQ in CP@NP‐cRGD was used to inhibit AZD9291‐induced autophagy by inhibiting autophagosome fusion with lysosomes, which leads to the aggregation of autophagosomes and increased LC3‐II levels. MDC staining was a specific indicator of autophagic vacuoles and was utilized to evaluate the level of autophagy in cells. Among all drug treatment groups, the use of rapamycin (a commonly used autophagy inducer) treatment as a positive control induced more green MDC puncta than control. Although untreated H1975/AR cells showed fewer green puncta, there was still basal autophagy occurred. Increased fluorescence intensity and some MDC‐labeled cells were monitored in AZD9291 and PN+AZD9291 treatment groups, suggesting AZD9291 and PD173074 induced autophagy (**Figure** **7**A). Results showed that more MDC‐labeled cells were observed in CP@NPs+AZD9291 and CP@NP‐cRGD+AZD9291 groups than cells treated with AZD9291 (Figure 7B), indicating that CQ inhibited drug‐induced autophagy in AZD9291‐resistant cells. The western blotting analysis revealed that LC3‐II levels were increased in AZD9291 and PN+AZD9291 groups, were significantly attenuated in the presence of CQ combination(in CN, FCP, CP@NPs, CP@NP‐cRGD group), and the CP@NP‐cRGD treatment yielded the most dramatic results (Figure 7C).

TEM is the gold standard technique to detect the presence of autophagy vesicles, which are typically identified as vacuolated structures surrounded by a thin film that insulates the contents of the cell. The arrow marked autophagosome formation in the enlarged image by TEM (Figure 7D). Compared with AZD9291 alone, AZD9291 plus CP@NP‐cRGD inhibited the degradation of autophagy, resulting in the most significant accumulation of autophagosomes.

mCherry–green fluorescent protein (GFP)–LC3B can be used to distinguish autophagosomes from autolysosomes. Due to the low lysosome pH value, the green fluorescence of GFP is quenching after the fusion of autophagosomes with lysosomes, while the effect of mCherry red fluorescence is not affected. Thus, in the merged image, yellow spots indicate autophagosomes, and red spots indicate autolysosomes. As shown in Figure 7E,F, in AZD9291 or PN+AZD9291 group, the formation of autophagosomes (yellow when combined) and autolysosomes (red when combined) increased, indicating that these two drugs enhanced the autophagy flux of cells. In CP@NPs or CP@NP‐cRGD group, the number of autophagosomes was significantly higher, and the number of autolysosomes decreased, suggesting that combined with CQ can inhibit the fusion of autophagosome with lysosome.

Collectively, these results indicated CP@NP‐cRGD could effectively inhibit autophagy, which is a key protective mechanism in resistant cells that respond to AZD9291.

### 2.6 Activity and Antitumor Efficiency of CP@NP‐cRGD in AZD9291‐Resistant NSCLC In Vivo

**Figure 8:**
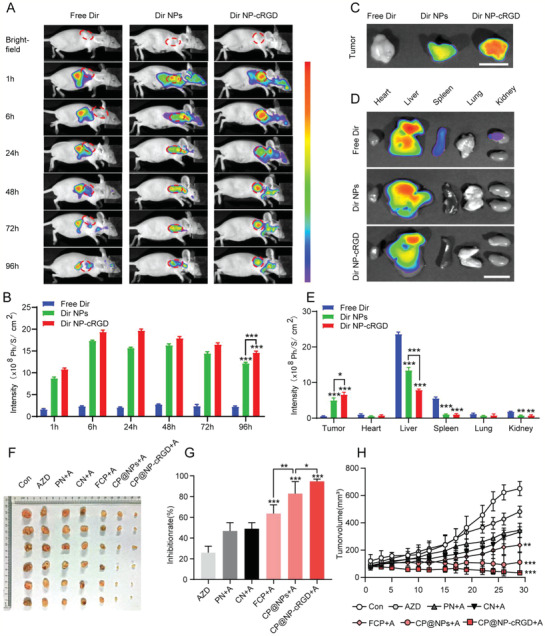
Activity and antitumor efficiency of CP@NP‐cRGD in AZD9291‐resistant NSCLC in vivo. A) In vivo distribution of free Dir, Dir NPs, and Dir NP‐cRGD in H1975/AR tumor‐bearing mice after intravenous injection for 1, 6, 24, 48, 72, and 96 h (*n* = 3). B) Quantitative average fluorescent intensities. C) Ex vivo fluorescence images of the dissected NSCLC tumors and D) normal organs 96 h after administration. Scale bar: 1 cm. E) Average fluorescent intensities. F) Images of AZD9291‐resistance subcutaneous xenograft tumors at the end of treatment. G) Tumor inhibition ratio and H) tumor growth curves of AZD9291‐resistant tumors during the treatment. All data were from three repeats, *p < 0.05, **p < 0.01, ***p < 0.001. A: AZD9291, PN: PD@NP‐cRGD, CN: CQ@NP‐cRGD, FCP: free chloroquine and PD173074.

To confirm the tumor‐targeting effect of NPs in vivo, the whole‐body distribution of Dir‐loaded NPs and cRGD‐NPs was determined by in vivo imaging (**Figure** **8**A). Free Dir, NPs‐Dir, and cRGD‐NPs‐Dir were delivered by caudal vein injection to nude mice, respectively. The selective accumulation of cRGD‐NPs and NPs in the tumor is faster and greater than that of the free drug, while the accumulation in the normal tissue is much less. Among them, cRGD‐NPs showed a more targeted distribution in vivo. Furthermore, it was demonstrated that a significant induction of bioluminescence at 1 h after treatment in cRGD‐NPs group, and enhanced till 96 h (Figure 8A,B). This data confirmed the efficacy of CP@NP‐cRGD with prolonged circulation time properties and suggested that daily treatment was not necessary to achieve sustained drug concentration. 96 h after the injection of the drug, we further confirmed the high tumor targeting efficiency of cRGD‐NPs by ex vivo imaging of the tumor (Figure 8C–E). The intensity of cRGD‐NPs in the tumor was higher than that of NPs, and there was almost no enrichment of free drugs in the tumor, suggesting that NPs could accumulate in tumor sites through EPR effect and cRGD has an active targeting ability. Besides, ex vivo imaging of organs showed that cRGD‐NPs and NPs were presented only in the liver, and the fluorescence intensity was much weaker than that of free Dir, which is found in the kidney, spleen, and liver (Figure 8D,E). These differences may imply that both NPs and cRGD‐NPs can exhibit little side effects on the organ.

To study the therapeutic efficiency of CP@NP‐cRGD on AZD9291‐resistant tumor in vivo, nude mice bearing AZD9291‐resistant NSCLC xenografts were used. First, consistent with our in vitro experiment, the tumor sizes of all the treatment groups were smaller than that of the control group, among which AZD9291 monotherapy showed a relatively weak antitumor effect (Figure 8F,G). As shown in Figure 8H, compared with the single‐targeted NPs group, dual‐targeted NPs groups (CP@NPs and CP@NP‐cRGD) exhibited slower growth, indicating the superiority of double‐targeted property.

The outstanding tumor‐targeting ability ensured the therapeutic effects of the dual‐targeted NPs group better than that of the dual‐targeted free drug group. In addition, the inhibition of tumor growth was most pronounced in CP@NP‐cRGD group, suggesting the better active targeting property of cRGD‐coated NPs. Taken together, CP@NP‐cRGD was demonstrated to effectively reverse AZD9291 resistance at best in vivo.

### 2.7 CP@NP‐cRGD Induced More Apoptosis and Less Proliferation by Inhibiting Protective Autophagy in AZD9291‐Resistant NSCLC In Vivo

**Figure 9:**
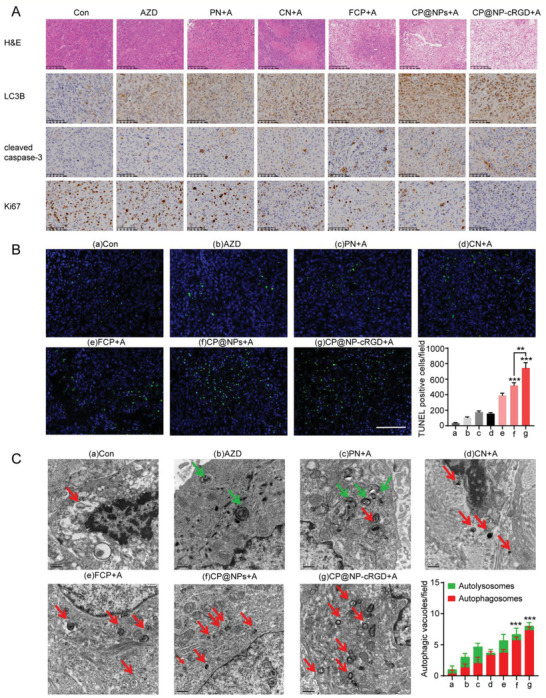
CP@NP‐cRGD induced more apoptosis and less proliferation by inhibiting protective autophagy in AZD9291‐resistant NSCLC in vivo. A) Tumor biopsies were stained with H&E, LC3B, cleaved caspase3, Ki67. The upper panels showed H&E results with 100‐fold magnification, and the lower panels showed IHC results with 200‐fold magnification. B) Apoptosis evaluation by TUNEL assay, representative TUNEL images with 200‐fold magnification and percentage of TUNEL‐positive cells. Scale bar: 100 µm. C) Representative electron microscopy images and quantitative analysis of autophagosomes (red) and autolysosomes (green) of tissue samples exacted from tumor xenografts.

Previous studies have shown that autophagy regulates both cell survival and cell death, and there is an interaction between autophagy and apoptosis.^[^ ^30^ ^]^ The effect of CP@NP‐cRGD on autophagy and apoptosis of AZD9291‐resistant cells in vivo was further investigated. Hematoxylin and eosin (H&E) analysis of tumor tissue indicated that the necrosis area in the CP@NP‐cRGD group was much larger than that in the other five groups (**Figure** **9**A). Immunohistochemical results showed that CP@NP‐cRGD could effectively increase apoptosis‐related proteins cleaved‐caspase3 and reduce the expression of proliferation‐related protein Ki‐67 (Figure 9A), consistent with in vitro results. pFGFR1 was also reduced by CP@NP‐cRGD (Figure S10, Supporting Information). Therefore, CP@NP‐cRGD can induce apoptosis and reduce the proliferation of AZD9291‐resistant cells potentially by inhibiting FGFR1 expression. Moreover, apoptosis was evaluated using terminal deoxynucleotidyl transferase dUTP Nick‐End labeling (TUNEL) staining. TUNEL assay showed a significant increase in apoptotic cells in tumor tissues in CP@NP‐cRGD group compared with the other five groups (Figure 9B). To examine the effect of CP@NP‐cRGD in the reversal of autophagy mediated AZD9291‐resistance in vivo, changes in the number of autophagy vesicles were observed in the tumor tissues by TEM (Figure 9C). TEM results displayed that there was few autophagosomes formation in control groups while AZD9291 group was characterized by massive vacuoles and autophagosomes accumulation. After combined with CQ, autophagosomes were further accumulated in AZD9291‐resistant cells. As revealed by immunohistochemistry assay, the amount of LC3B puncta was much higher in the single‐AZD9291 group than the controls (Figure 9A), and this increase was aggravated by CP@NP‐cRGD. This enhanced autophagosome accumulation may be the result of increased autophagosome formation (autophagy induction) and decreased autophagosome turnover (autophagy arrest). To sum up, these results suggested that by simultaneous inhibition of the FGFR1 pathway and autophagy, CP@NP‐cRGD could sharply induce apoptosis and decrease proliferation in AZD9291‐resistant NSCLC tumor in vivo.

### 2.8 Efficient Evasion of Drugs Side Effects of CP@NP‐cRGD In Vivo

**Figure 10:**
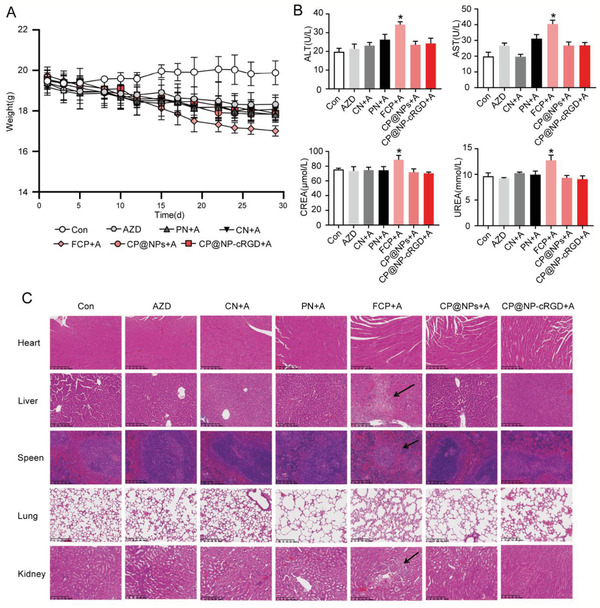
In vivo side effects evaluation. A) Body weight curves of mice in different drug groups during the experimental period. B) Hepatic (ALT and AST) and renal (UREA and CREA) function of nude mice after the last drug administration. All data were from three repeats. C) Histological analysis of major organs (heart, liver, spleen, lung, and kidney) from nude mice (Scale bar: 200 µm).

To detect the toxicity of CP@NP‐cRGD in vivo, the following experiment was done. First, we plotted the bodyweight curve of these nude mice and evaluated the systemic toxicity of different treatment groups. The total bodyweight of the free CQ/PD group decreased significantly, suggesting that free drugs may have more systemic toxicity (**Figure** **10**A). Levels of alanine aminotransferase (ALT), aspartate aminotransferase (AST), creatinine (CREA), and urea in the serum of all the NP groups were in the normal range while the free CQ/PD group had mildly increased levels (Figure 10B), suggesting the hepatic and renal toxicity of these free drugs. CP@NP‐cRGD or other NPs treatments did not show weight loss and significant toxicity. To further clarify the potential toxicity toward major organs, H&E analysis of major organs (heart, liver, spleen, lung, and kidneys) was performed four weeks later since the first treatment. Except for the free CQ/PD group, no significant liver, kidney damage, lung toxicity, heart damage, or inflammatory infiltration of the spleen were observed in the NPs group (Figure 10C). In the free CQ/PD group, there were significant liver necrosis and a small amount of inflammatory infiltration of the spleen and kidney.

## 3 Discussion

The incidence of lung adenocarcinoma has increased significantly over the past 20 years, especially among females.^[^ ^31^ ^]^ The third‐generation EGFR‐TKI AZD9291 has significantly improved survival, reaching 38.6 months of median overall survival. The encouraging data is resulting from its high efficacy and low toxicity; however, its resistance is inevitable.^[^ ^5b^ ^]^ The mechanisms of resistance to AZD9291 are heterogeneous, with a higher proportion of EGFR‐independent pathways than EGFR‐dependent reasons.^[^ ^32^ ^]^ Currently, patients have limited options and combinations therapy is commonly used to overcome AZD9291 resistance. For example, AZD9291 was reported to be combined with first‐generation EGFR‐TKI, MEK inhibitors, or RET inhibitors and achieved good efficacy in patients with AZD9291 resistance related to EGFR C797S transmutation, MET amplification, or RET fusion, respectively.^[^ ^33^ ^]^ Autophagy is a highly conserved catabolic process and plays a controversial role in EGFR‐TKI resistance. Lower sensitivity to erlotinib treatment in NSCLC cells can be restored by combining erlotinib with autophagy‐inducing drug rapamycin.^[^ ^34^ ^]^ While targeting AXL can abrogate autophagic flux and induce immunogenic cell death in EGFR‐TKI resistant cancer cells.^[^ ^35^ ^]^ In this study, we identified the different autophagy levels between AZD9291‐resistant and AZD9291‐sensitive cell lines with both EGFR sensitive and T790M mutation, suggesting a protective role of autophagy in AZD9291 treatment. Furthermore, we found that autophagy‐related protein levels regulated by AZD9291 were dose‐ and time‐dependent in NSCLC cells.

FGFR1 is a receptor tyrosine kinase that plays a crucial role in a variety of biological processes in tumor cells, including survival, proliferation, apoptosis, and differentiation.^[^ ^36^ ^]^ FGFR1 signaling has a close relationship with EMT‐associated resistance of 1st and 2nd EGFR‐TKIs.^[^ ^18b,c^ ^]^ Recently, focal FGFR1 amplification was observed in the AZD9291‐resistant tumor from one EGFR‐T790Mmutant NSCLC patient.^[^ ^37^ ^]^ Our data indicated that FGFR1 overexpression might also contribute to AZD9291 resistance. Interestingly, inhibition of FGFR1 not only restored the sensitivity of AZD9291‐resistant cells but also induced autophagy in these cells. Inhibition of FGFR1 could induce autophagy in FGFR1‐amplified lung squamous cell carcinoma cells, and simultaneously blockade of both FGFR1 and autophagy could enhance cell death.^[^ ^25^ ^]^ These findings suggested that autophagy and FGFR1 inhibitor may be promising AZD9291‐sensitizers, and simultaneous inhibition of autophagy and FGFR1 on AZD9291 resistant NSCLC cells could potentially enhance the efficacy of AZD9291 treatment. CQ was used to inhibit autophagy in our study because it is an FDA‐approved and clinically widely‐used medicine. In addition, CQ could normalize tumor vessel structure to improve tumor hypoxia and intratumor chemotherapeutic delivery and response.^[^ ^38^ ^]^ CQ can also facilitate endosomes' escape of nanocomplexes and result in enhanced transfection efficiency.^[^ ^12^, ^39^ ^]^ Actually, CQ or its derivative hydroxy‐CQ is being tested clinically as an autophagy inhibitor in combination with many anticancer agents. However, the potential systemic toxicity and limited drug absorption in vivo restrict the application of CQ in clinical tumor therapy.^[^ ^40^ ^]^ To design an agent with a long administration period and low side effects to reverse AZD9291 resistance, we designed a multifunctional nanocarrier delivery system CP@NP‐cRGD that dual‐targeting FGFR1 pathway and autophagy. Specifically, the pH‐sensitive CaP shell can avoid drug inactivation through lysosomal escape, and firstly reduce autophagy level through CQ release, and then release PD173074 to inhibit the FGFR1 pathway. Then, the exposed PEG hydrophilic chain forms a hydration film to maintain long cycle stability. At last, with an appropriate size, EPR effect, and cRGD peptide, CP@NP‐cRGD can accurately deliver the drug to the tumor tissue, achieving good dual‐tumor targeting. Consequently, CP@NP‐cRGD was shown to potently inhibit autophagy and FGFR1 pathways to overcome AZD9291 resistance in NSCLC cell lines with optimal drug protection and sequential release ability. Furthermore, in vivo results showed that combining CP@NP‐cRGD with AZD9291 significantly decreased the growth of NSCLC AZD9291 resistance‐tumor xenografts and exhibited excellent targeting properties, sustained releasing potential, good stability, and low toxicity. Therefore, CP@NP‐cRGD may be an efficient and relatively safe option agent for overcoming AZD9291 resistance. The ERK1/2 signaling pathway is a receptor tyrosine kinase‐mediated signaling pathway involved in a variety of cellular processes, such as proliferation, cell cycle, and drug resistance. In this study, CP@NP‐cRGD showed a significant inhibitory effect of ERK1/2 phosphorylation in the AZD9291‐resistant NSCLC cell line, while there was no significant difference in t‐ERK1/2 level, suggesting the involvement of the ERK1/2 pathway of AZD9292‐resistance, which need further study.

In sum, this study was undertaken to design a multifunctional NP CP@NP‐cRGD and evaluate its role in antagonizing AZD9291 resistance. The results suggested that CP@NP‐cRGD had the potential to replace free drug combinations in clinical therapies and was required for future in vivo testing.

## 4 Experimental Section

##### Establishment of AZD9291‐Resistant Cell Lines

Lung adenocarcinoma cell lines H1975 and HCC827 were purchased from the American Type Culture Collection (ATCC, Manassas, VA, USA). Cell lines were grown in RPMI‐1640 medium supplemented with 10% fetal bovine serum. Cell identity was evaluated using short tandem repeat profile analysis by Biowing Biotechnology Co., Ltd. (Shanghai, China), the method was described in 2012 in an ANSI Standard (ASN‐0002) by the ATCC Standards Development Organization. Cells were routinely tested for mycoplasma contamination using mycoplasma‐specific primers and were found to be negative. The establishment method of AZD9291‐resistant NSCLC cell lines was the same as the previous reports.^[^ ^41^ ^]^ Briefly, AZD9291‐resistant H1975 and HCC827 cell lines (H1975/AR and HCC827/AR) were established by a stepwise increase in the concentrations of AZD9291 for 72 h with a recovery period between treatments. About six months later, H1975/AR and HCC827/AR cell lines were successfully established.

##### Synthesis and Characterization of CP@NP‐cRGD

cRGD‐PEG‐DSPE was synthesized by coupling the targeted ligand c(RGDfC) (Meiluo Technology Co., Ltd., Shenzhen, China) with the COOH group of COOH‐PEG2000‐DSPE(Meiluo Technology Co., Ltd., Shenzhen, China) using EDC as a coupling agent. Samples were dissolved in deuterated chloroform (CDCl3), and ^1^H NMR spectra were recorded on Bruker Avance 500 (500 MHz) spectrometer (Beijing, China). PD173074 (Selleckchem, S1264) loaded cRGD‐PEG‐DSPE micelles were freshly prepared using the thin‐film hydration method. CQ (Sigma‐Aldrich, C6628) and calcium chloride water solution were then mixed with the prepared micelles solution. Next, HBS (Hepes, NaCl, Na_3_PO_4_, pH 7.4) buffer solution was immediately added to the above mixture. The solution was left to stand for 30 min to get CP@NP‐cRGD. The final concentration of PD173074 was 10 µg mL^−1^, and the final concentration of CQ was 200 µg mL^−1^; a 20‐fold dilution was used in cell experiments. Rhodamine B (RB) or 1,1‐dioctadecyl‐3,3,3,3‐ tetramethylindotricarbocyaine iodide (Dir) might replace PD173074 to prepare NPs‐Dir and NPs‐RB for different experiments.

##### Physical and Chemical Properties of CP@NP‐cRGD

The size and surface zeta potential of NPs were detected using the Zetasizer Nano ZS90 size analyzer (Malvern Panalytical, Malvern, England). The morphology of the NPs was measured using a TEM (Talos F200X, Shanghai, China). Samples were dropped on copper grids coated with a layer of carbon and dried at room temperature. Drug loading capacity and entrapment efficiency of PD173074 and CQ in CP@NP‐cRGD were determined after ultracentrifugation by HPLC. Drug loading content was determined as the mass ratio of the drug content to the NPs. The drug encapsulation efficiency was defined as the ratio of the drug content in the NPs to the initial drug amount added for the NPs. The concentrations of CQ and PD173074 were diluted to 10 and 0.5 µg mL^−1^, respectively.

##### Western Blot Analysis

The cell lysate was prepared using RIPA Buffer (Beyotime, Shanghai, China, P0013B) with a protease and phosphatase inhibitor cocktail (Sigma‐Aldrich, PPC1010). Samples were subjected to SDS‐PAGE, transferred to polyvinylidene difluoride B membranes, blocked by 5% nonfat milk in TBST, and sequentially incubated with the indicated primary antibodies. For detection, HRP‐conjugated secondary antibodies (Cell Signaling, Beverly, MA) and chemiluminescent HRP substrate kit (Merck Millipore) were used. The following primary antibodies were used: anti‐LC3B (Abcam, ab51520), anti‐SQSTM1 (CST, 8025), anti‐FGFR1 (CST, 9740), anti‐pFGFR1 (CST, 52928), anti‐ERK (CST, 4659), anti‐pERK (CST, 4370), anti‐survivin (CST, 2803), anti‐cleaved‐caspase3 (CST, 9664), and anti‐caspase3 (CST, 9662).

##### Cell Proliferation Assays

Cell proliferative capacity was measured using CCK8 (Dojindo, Japan) and clone formation assays. For the CCK‐8 assay, cells were incubated in 96‐well plates in the presence of different drugs for 24, 48, or 72 h. Then, a 10 µL of CCK‐8 solution was added per well. The plates were incubated for 30 min at 37 °C, with the results read at an optical density of 450 nm. IC50 values were calculated using GraphPad Prism 8.0 software (La Jolla, CA, USA). For the clone formation assay, 100 cells per well were seeded in 6‐well plates with different treatment for two weeks. Colonies were fixed by 4% paraformaldehyde and stained by 2.5% crystal violet.

##### Hoechst/PI Staining

The apoptosis/necrosis assay kit (Beyotime, Shanghai, China, C1056,) was used to simultaneously monitor apoptotic, necrotic, and healthy cells. Cells were washed with cold PBS three times, incubated with 1 mL of binding buffer, 5 µL Hoechst solution, and 5 µL propidium iodide (PI) solution for 30 min, and analyzed by fluorescence microscopy. Apoptotic cells were Hoechst‐positive and PI‐negative, and necrotic cells were Hoechst and PI‐positive.

##### Drug Release of CP@NP‐cRGD

The drug release study was performed in different pH mediums (5.5 and 7.4). The release profile of PD173074 and CQ in vitro was evaluated using the dialysis method. The dialysis tube was placed in a tube containing 20 mL of release media (PH = 5.5 or 7.4) and then placed in a shaking bath at 37 °C. 0.2 mL of the release sample was collected at each time point and replaced with an equal amount of fresh release medium. The drug content of CQ or PD173074 released into the medium was calculated by HPLC and compared with the generated standard calibration curve.

##### Cellular Uptake of cRGD‐CaP NPs

Cells were incubated in free RB (Sigma‐Aldrich, R6626)(20 µg mL^−1^), RB NPs (20 µg mL^−1^), and RB NP‐cRGD (20 µg mL^−1^) medium respectively for 1 and 4 h, then were washed three times with PBS, labeled the nucleus with 1 mL Hoechst 33342 (Beyotime, Shanghai, China, C1025) and imaged by confocal laser scanning microscopy (Olympus, Japan, FV1000). Furthermore, cells were harvested, and RB accumulation was analyzed by flow cytometry (Becton Dickinson, USA).

##### Flow Cytometry

Flow cytometric assay was used for apoptosis and cell cycle analysis. For apoptosis analysis, cells were collected and stained with Annexin V‐FITC/PI (556547, BD Biosciences, Franklin Lakes, USA) at room temperature in the dark and then analyzed. The percentage of Annexin V‐positive/PI‐negative (early apoptosis) and Annexin V‐positive/PI‐positive (late apoptosis) cells were calculated according to the manufacturer's instruction. For cell cycle analysis, cells were seeded in 6‐well plates and incubated with different drugs. 48 h after treatment, cells were washed with PBS and fixed in 70% ethanol at 4 °C overnight and then stained with PI. Cells were analyzed by flow cytometer (Becton Dickinson, USA) and quantified using ModFit 3.2 software (Verity Software House, Topsham, ME, USA).

##### Ad‐mCherry–GFP–LC3B Transfection Assays

Cells were cultured to 50–60% confluence and transfected with Ad‐mCherry–GFP–LC3B adenovirus (Beyotime, C3011) at an MOI of 30 at 37 °C for 24 h. It is a recombinant adenovirus, which can effectively express fusion proteins of red fluorescent protein mCherry, GFP, and LC3B in target cells after infection. In the case of nonautophagy, mCherry–GFP–LC3B exists in the cytoplasm in the form of diffuse yellow fluorescence (the combined effect of mCherry and GFP). In the case of autophagy, mCherry–GFP–LC3B aggregated on the autophagosome membrane, showing yellow spots. When the autophagosome fuses with the lysosome, the portion of the GFP fluorescence is quenched and appears as a red spot. Following AZD9291 and NPs treatment, LC3B puncta were recorded with a fluorescence microscope and quantified with the ImageJ program (NIH, Bethesda, MD, USA).

##### TEM

TEM was performed to observe autophagic vacuole ultrastructure as described before.^[^ ^42^ ^]^ NSCLC cells or tissue samples (≈1 mm^3^) were fixed in 2.5% glutaraldehyde at 4 °C overnight and washed with PBS twice and fixed in osmic acid for 2 h. Then samples were dehydrated by gradients of ethyl alcohol and acetone, embedded and saturated, sliced into ultrathin sections. Ultrathin sections were stained with uranyl acetate and lead citrate and observed with an EM420 transmission electron microscope (Philips, Eindhoven, The Netherlands).

##### MDC Staining

MDC (Sigma‐Aldrich, 30432) is used as a selective marker for autophagic vacuoles and especially autolysosomes. MDC staining was then analyzed by flow cytometry and fluorescence microscope. Cells were cultured in 6‐well plates and treated with AZD9291, alone or combined with NPs. After 48 h of treatment, cells were exposed to MDC (50 µmol) for 20 min at 37 °C in the dark. After incubation, cells were washed twice in PBS 1× and examined using a fluorescence microscope (Olympus IX51, Tokyo, Japan), or collected and analyzed by flow cytometer.

##### Xenografts and Treatments

All of the procedures, including tumor transplantation, drug delivery, and euthanasia, were approved by the Ethics Committee of Shanghai Chest Hospital (KS2034). For the subcutaneous xenograft model, female BalB/C nude mice (6 to 8‐week‐old, weighing 18–20 g) were maintained under an SPF environmental condition. Briefly, H1975/AR cells at the density of 1  ×  10^7^ were subcutaneously injected in the right flanks of each mice. The nude mice were randomly divided into seven groups, and each group contained six mice. The groups were intravenously administered respectively with saline (con and AZD groups), PD@NP‐cRGD (PN group), CQ@NP‐cRGD (CN group), free CQ/PD (FCP group), CQ/PD@NPs (CP@NPs group), and CQ/PD@NP‐cRGD (CP@NP‐cRGD group), at the dose of CQ 20 and PD173074 1 mg kg^−1^ twice a week for four weeks. AZD9291 (5 mg kg^−1^) was given via gavage every day except the control group.

##### Distribution of cRGD‐CaP‐NPs In Vivo

Dir (40757ES25,Yeasen, Shanghai, China), a hydrophobic fluorescence label, was encapsulated into CaP‐NPs and cRGD‐CaP‐NPs. Free Dir, Dir‐NPs, and Dir‐NPs‐cRGD were delivered by intravenous injection, and their biodistribution in mice was studied using an in vivo imaging system (Berthold Technologies Gmbh & Co. KG, Bad Wildbad, Germany, LB983). At 96 h post‐injection, the mice were sacrificed, and the individual organs and tumors were harvested and scanned. The images were analyzed by indiGo software.

##### Antitumor Efficacy In Vivo

Tumor volume was measured three times a week until the animals were sacrificed and calculated according to the formula, *V* = *ab* ^2^/2 (a represented the length and b was the width). The tumor growth curve was drawn, and the inhibition rate of the tumor was calculated. TUNEL assay was used to detect apoptosis of tumor tissues, using the DeadEnd Fluorometric TUNEL System (Promega, Madison, WI, G3250) according to the manufacturer's instructions.

##### Immunohistochemistry

The expression levels of p‐FGFR1, LC3B, cleaved‐caspase3, and Ki67 in tumor tissues were detected by immunohistochemistry (IHC). In simple terms, the paraffin sections were deparaffinized, rehydrated, and washed. Then tissue sections were preincubated with 3% hydrogen peroxide and blocked with nonfat milk. The sections were incubated with primary antibody at 4 °C overnight. Then each section was washed with PBS, and the secondary antibody was incubated. The tumor sections were counterstained with hematoxylin, and observed by a microscope (Zeiss, Germany).

##### Toxicology Evaluations

The body weight of all nude mice was measured three times a week. After treated with various drugs, the toxic effects of NPs on major organs of mice (kidney, spleen, liver, lung, and heart) were determined by H&E staining. Histological observations were carried out by a microscope (Zeiss, Germany). Furthermore, whole blood samples were harvested, and a blood routine examination was performed using an automatic blood cell analyzer (Kangyu Medical Instruments Co., Ltd., Guangzhou, China, HF‐3800). Liver and kidney functions were accessed using serum samples by specific assay kits (Nanjing Jian Cheng Institute, Nanjing, China, C009‐2‐1, C0010‐2‐1,C013‐2‐1,C011‐2‐1).

##### Statistical Analysis

Statistical analyses were performed using SPSS software (version 21.0, IBM Corp., Armonk, NY, USA). Data were reported as the mean ± SD. Statistical comparison was assessed by one‐way ANOVA. Multiple comparisons were performed by Tukey's post hoc test or Sidak's post hoc test. All statistically processed results were determined to be significant at *p* < 0.05. *p < 0.05, **p < 0.01, ***p < 0.005.

## Conflict of Interest

The authors declare no conflict of interest.

## Supporting information

Supporting Information
