# Supplementary material for: AZD9291 Resistance Reversal Activity of a pH‐Sensitive Nanocarrier Dual‐Loaded with Chloroquine and FGFR1 Inhibitor in NSCLC
Source: Adv Sci (Weinh). 2020 Dec 4;8(2):2002922. doi: 10.1002/advs.202002922 (PMC7816715; doi:10.1002/advs.202002922)
Supplement: Supplementary file 1 — Supporting Information [file ADVS-8-2002922-s001.pdf]

WILEY-VCH

© 2020 Wiley-VCH GmbH

ADVANCED SCIENCE
Open Access

Supporting Information

for *Adv. Sci.*, DOI: 10.1002/advs.202002922

AZD9291 Resistance Reversal Activity of a pH-sensitive nanocarrier dual-loaded with chloroquine and FGFR1 inhibitor in NSCLC

Yu Gu, Songtao Lai, Yang Dong, Hao Fu, Liwei Song, Tianxiang Chen, Yourong Duan*, Zhen Zhang**

WILEY-VCH

© 2020 Wiley-VCH GmbH

Supporting Information

AZD9291 Resistance Reversal Activity of a pH-sensitive nanocarrier dual-loaded with chloroquine and FGFR1 inhibitor in NSCLC

Yu Gu, Songtao Lai, Yang Dong, Hao Fu, Liwei Song, Tianxiang Chen, Yourong Duan*, Zhen Zhang**

Supporting Information

AZD9291 Resistance Reversal Activity of a pH-sensitive nanocarrier dual-loaded with chloroquine and FGFR1 inhibitor in NSCLC

Yu Gu, Songtao Lai, Yang Dong, Hao Fu, Liwei Song, Tianxiang Chen, Yourong Duan*, Zhen Zhang**

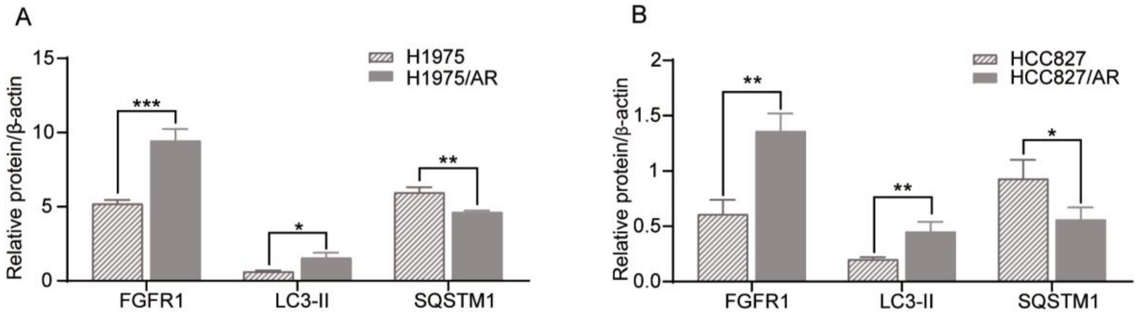

Figure S1. Quantitative grayscale intensity of protein expression in parental and AZD9291 resistant cells(H1975 and HCC827 cell lines). Values were normalized to β -actin protein levels. *P<0.05, **P<0.01, ***P<0.001.

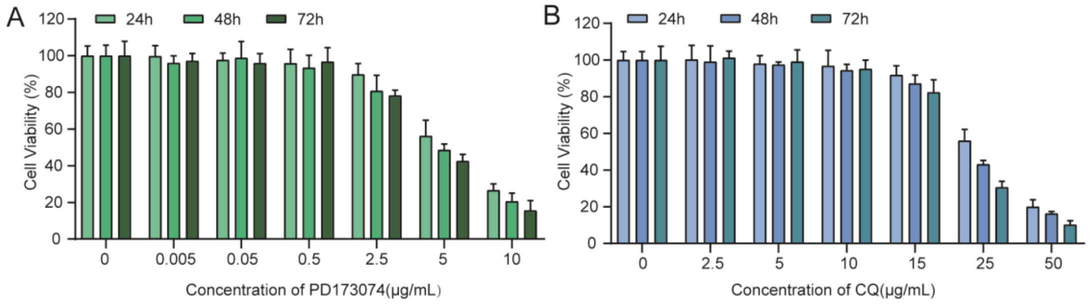

Figure S2. *In vitro* toxicity of PD173074 and CQ. Cell viability of AZD9291-resistant cells H1975/AR treated with different concentrations of PD173074(A) and CQ(B) in 72h.

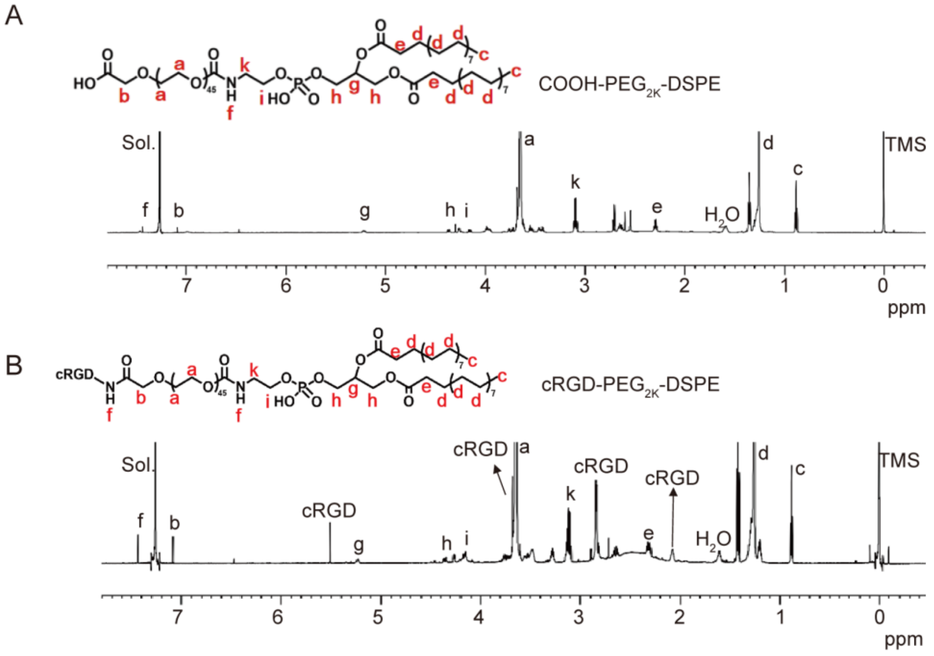

Figure S3. The ^{1}H NMR spectra of the DSPE-PEG$_{2k}$-COOH (A) and DSPE-PEG$_{2k}$-cRGD(B) in $CDCl_3$. The characteristic peaks were pointed out.

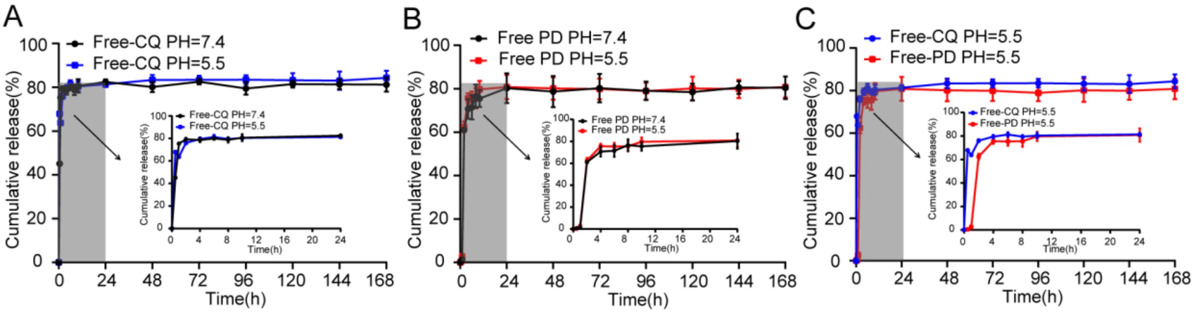

Figure S4. Release profile of free CQ(A) and free PD173074(B) in pH 5.5 and 7.4. CQ and PD173074 were released simultaneously from free CQ-PD in pH 5.5(C). Each data was expressed as mean±standard deviation (n=3).

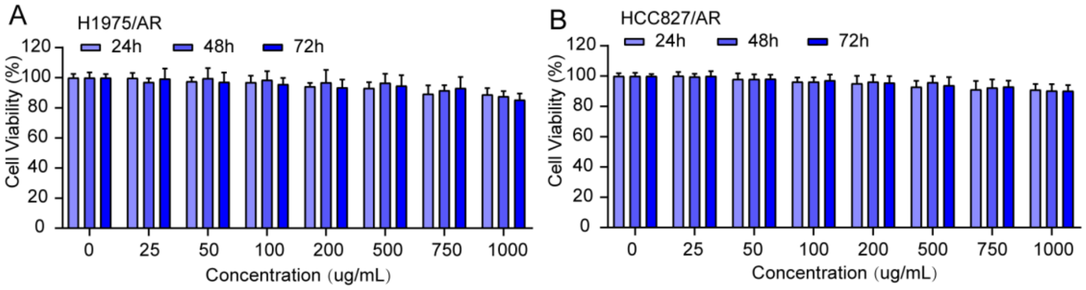

Figure S5. *In vitro* toxicity of cRGD-CaP NPs. Cell viability of H1975/AR(A) and HCC 827/AR(B) treated with different concentrations of cRGD-CaP NPs in 72h.

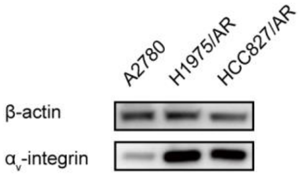

FigureS6. Western blot analysis showing the expression of integrin protein of different cell lines. Integrin α v- negative A2780 human ovarian tumor cells as a negative control.

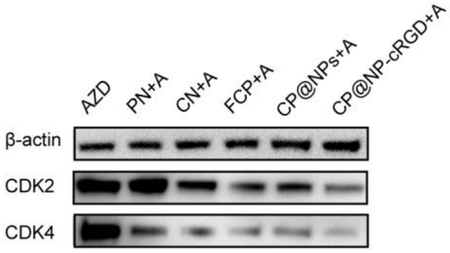

Figure S7. Western blot analysis showing the expression of cell cycle-associated proteins after different treatments.

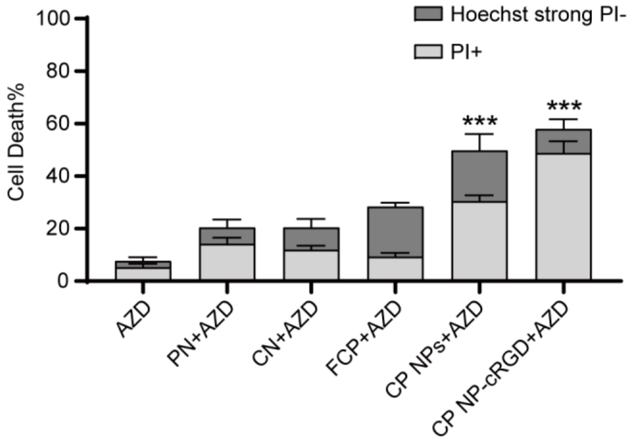

Figure S8. Quantification of cell death (apoptosis and necrosis) of different treatments in H1975/AR cells. All data are from five fields, ***p<0.001, compared with AZD9291 alone group.

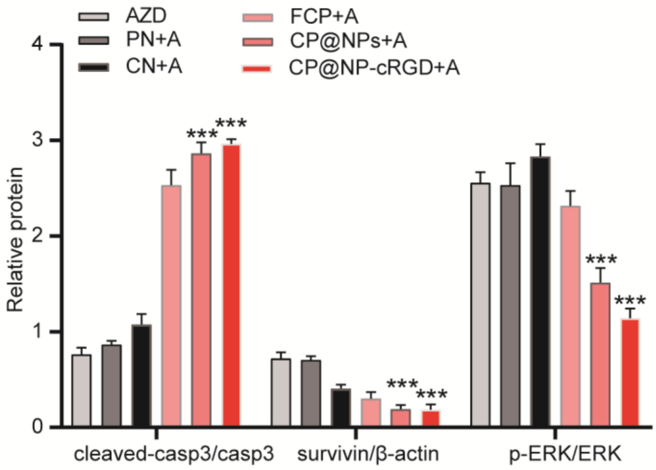

Figure S9. Quantitative grayscale intensity of protein expression of different treatments in H1975/AR cells. All data are from three repeats. Values are normalized to β-actin protein levels. ***P<0.001, compared with AZD9291 alone group.

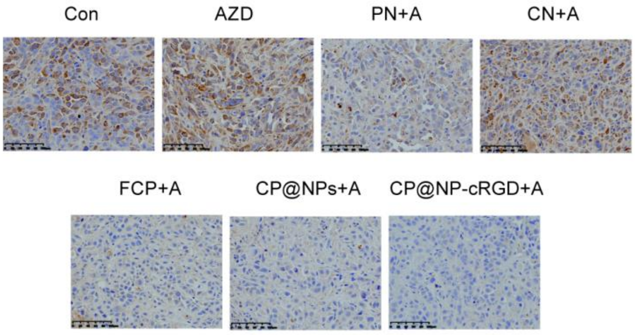

Figure S10. Immunohistochemistry results of proteins pFGFR1 with 200-fold magnification (At scale 100μm).
